# Reducible burden of laryngeal cancer in men aged 50 and older attributable to smoking and alcohol use: insights from the global burden of disease study 2021

**DOI:** 10.3389/fpubh.2025.1577138

**Published:** 2025-06-09

**Authors:** Ru Chen, Jing Deng, Yao Sun, Dongxun Sun, Haibin Lu, Guoqi Sima

**Affiliations:** Department of Otolaryngology, The First Hospital of Jiaxing, The Affiliated Hospital of Jiaxing University, Jiaxing, China

**Keywords:** laryngeal cancer, smoking, alcohol use, men, global burden of disease

## Abstract

**Background:**

Smoking, alcohol use, advanced age, and male gender are well-established risk factors for laryngeal cancer. As smoking and alcohol consumption are modifiable behaviors, the burden of laryngeal cancer attributable to these factors is potentially reducible. This study aims to quantify the global, regional and national burden of laryngeal cancer attributable to smoking and alcohol use in men aged 50 years and older, assess temporal trends from 1990 to 2021, and project the disease burden through 2040.

**Methods:**

The burden of laryngeal cancer was assessed using data from the Global Burden of Disease 2021, including deaths, disability-adjusted life years (DALYs), years of life lost (YLLs), and years lived with disability (YLDs). The estimated average percentage change (EAPC) was used to evaluate trends in the disease burden from 1990 to 2021. Finally, projections for 2040 were generated and calculated with Nordpred.

**Results:**

From 1990 to 2021, globally, the absolute burden of laryngeal cancer—measured by deaths, DALYs, YLLs, and YLDs—attributable to smoking and alcohol use increased among men aged 50 years and older, despite declining age-standardized rates (ASRs). Regionally, Central and Eastern Europe experienced the highest burden, which may be attributed to historically elevated rates of smoking and alcohol consumption in these regions. Countries with the greatest disease burden related to smoking at the national level include Cuba, Montenegro, Seychelles, Georgia, and Monaco, while Montenegro, Bulgaria, Romania, and Monaco have the highest alcohol-related burden. By 2040, the overall burden is projected to rise.

**Conclusion:**

Smoking and alcohol-related laryngeal cancer will pose a significant public health challenge in the future. As the aging demographic continues to grow, the disease burden among older adult men is expected to rise. There are notable regional and national differences in terms of deaths, DALYs, YLLs, and YLDs. To address the rising burden, targeted public health actions are urgently required. These include higher taxation on tobacco and alcohol, comprehensive bans on related advertising, stricter enforcement of sales and age restrictions, enhanced health education regarding their health risks, and strengthened efforts in early screening.

## Introduction

Laryngeal cancer is the most common malignant tumor in the head and neck, upper digestive tract, and respiratory system (1). Due to the functional importance of the larynx in phonation, respiration, and swallowing, common clinical symptoms in patients with laryngeal cancer include hoarseness, dyspnea, and dysphagia (2). In 2021, the global age-standardized incidence rate (ASIR) of laryngeal cancer was estimated at 2.29 per 100,000 population (95% uncertainty interval [UI]: 2.13–2.47), and approximately 100,000 deaths were reported annually worldwide (3, 4), resulting in substantial disability-adjusted life years (DALYs) lost and imposing a significant burden on society (5). Given its multifactorial etiology, understanding the key risk factors is essential for better mitigating the disease burden.

Smoking, alcohol use, advanced age, and male gender are well-established risk factors for laryngeal cancer (5, 6). Among these, smoking and alcohol consumption are modifiable behavioral risk factors, as they are preventable through behavioral or policy interventions. These modifiable exposures are especially relevant in adults over 50, where accumulated use increases cancer risk and is closely linked to higher cancer mortality (7, 8). For laryngeal cancer specifically, epidemiology-based studies indicate that incidence, mortality, and DALYs are predominantly concentrated among individuals aged 50 years and older (9–11), highlighting age 50 as a clinically relevant threshold in laryngeal cancer burden analysis. In this study, we define the “reducible burden” as the portion of disease burden theoretically preventable through effective control of key modifiable risk factors, particularly smoking and alcohol consumption. Despite the importance of addressing these factors in older adults, few studies have systematically examined the burden of laryngeal cancer in this demographic or quantified the share attributable to them.

Previous global studies have examined the overall burden of laryngeal cancer (4, 5), however, most analyses pooled data across all ages and sexes, limiting insights into high-risk groups like older men. The role of modifiable behavioral risk factors also remains underexplored. Although one study included smoking- and alcohol-related risk factors, it lacked age-specific data for men aged 50 and above—a key demographic with a disproportionately high burden of laryngeal cancer (12). These gaps hinder the development of targeted interventions for older male populations.

To address these gaps, this study focuses on men aged 50 years and older and quantifies the burden of laryngeal cancer attributable to smoking and alcohol use. Using Global Burden of Disease (GBD) 2021 data, we assessed mortality, DALYs, years of life lost (YLLs), and years lived with disability (YLDs) from 1990 to 2021, and explored variations by region, country, and socio-demographic index (SDI). By analyzing these long-term trends and disparities, we aim to generate evidence that supports targeted prevention strategies and informs policy decisions—such as optimizing resource allocation and strengthening tobacco and alcohol control in high-burden populations.

## Methods

### Data sources

GBD 2021 offers extensive data on 371 diseases and 88 risk factors, covering the global level, 21 regions, and 204 countries. This study primarily analyzes laryngeal cancer (due to alcohol use and smoking) among male patients in high-risk age groups, with high-risk age defined as individuals aged 50 and above. In GBD 2021, laryngeal cancer refers to malignant neoplasms of the larynx and is categorized under ICD-10 codes C32. Smoking typically refers to any form of tobacco use, while alcohol use typically refers to any form of alcohol consumption. Using the retrieval function of the Global Health Data Exchange, data can be filtered based on various parameters such as gender, age, disease type, SDI, region, and country.

### Estimation framework

The GBD framework computes health metrics based on the detailed methodologies outlined earlier (13–15). DALYs are used to measure the years of life lost due to disability and premature death, providing an overall indicator of the health loss caused by a disease from its onset to death. YLDs reflect the years lost to disability caused by the disease, while YLLs indicate the years of life lost from early death due to disease.

### Socio-demographic index

The SDI serves as a crucial composite measure of development status in the GBD. It integrates various factors such as mean educational attainment, income per person, and birth rates. The SDI spans from 0 to 1, where higher values signify more advanced levels of development. SDI quintiles divide nations and regions into five categories, facilitating comparisons of development levels and health outcomes across different areas.

### Statistical analysis

Statistical analysis was performed with R software (version 4.2.2). Mortality, DALYs, YLLs, and YLDs (both numbers and ASRs per 100,000) were used to evaluate the burden of laryngeal cancer linked to smoking and alcohol consumption. The uncertainty of the data was quantified using 95% uncertainty intervals (UI). A log-linear regression model was used to calculate the estimated annual percentage change (EAPC) for each indicator, reflecting trends in the data from 1990 to 2021 (16). For inequality analysis based on DALYs, it is advised to use the Slope Index of Inequality (SII) and the Concentration Index to evaluate both absolute and relative income disparities across countries (17). The decomposition analysis divided DALYs into three factors—aging, population, and epidemiology—to illustrate their impact on health disparities and the overall health burden (18). Frontier analysis examines potential health improvements that can be achieved based on the current development level across 204 countries (19). The Nordpred forecasting model was employed to predict burdens of laryngeal cancer in high-risk age group men up to the year 2040. The Nordpred model integrates the characteristics of the age-period-cohort framework, decomposing the time dimension into age, period, and cohort effects (20). It has been optimized and improved to more accurately predict future disease trends and is widely recognized in academic discussions.

## Results

### Global burden of laryngeal cancer attributed to smoking and alcohol use

**Figure 1:**
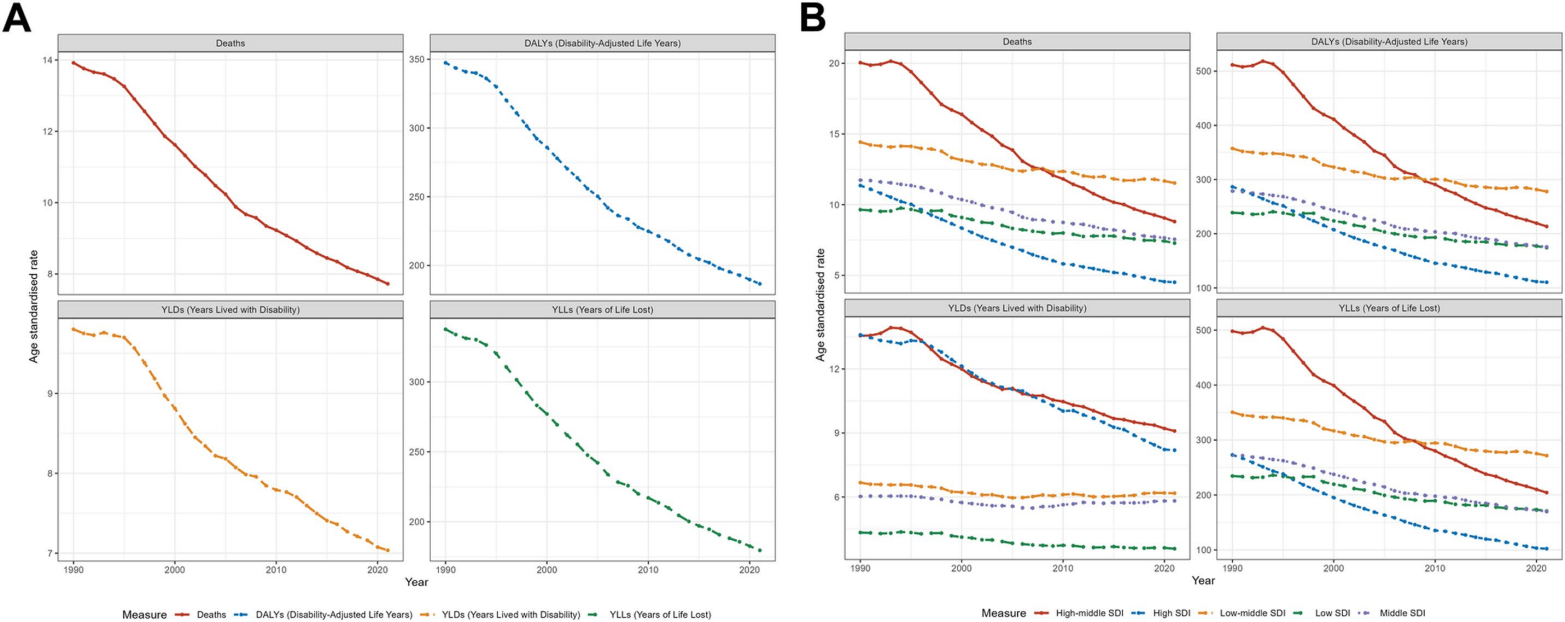
Trends in laryngeal cancer deaths, DALYs, YLDs, and YLLs from 1990 to 2021: **(A)** at the global level and **(B)** across different SDI levels, focusing on men over 50 years old and cases caused by smoking.

From 1990 to 2021, the burden of laryngeal cancer due to smoking in men over 50 increased in terms of deaths, DALYs, YLDs, and YLLs. However, the corresponding ASRs all decreased (Figure 1A). In 2021, low-middle SDI regions had the highest ASRs for deaths, YLLs, and DALYs, while high-middle SDI regions had the highest ASRs for YLDs (Figure 1B). Detailed data are provided in Supplementary Tables S1, S2 presents the trends in disease burden from 1990 to 2021.

**Figure 2:**
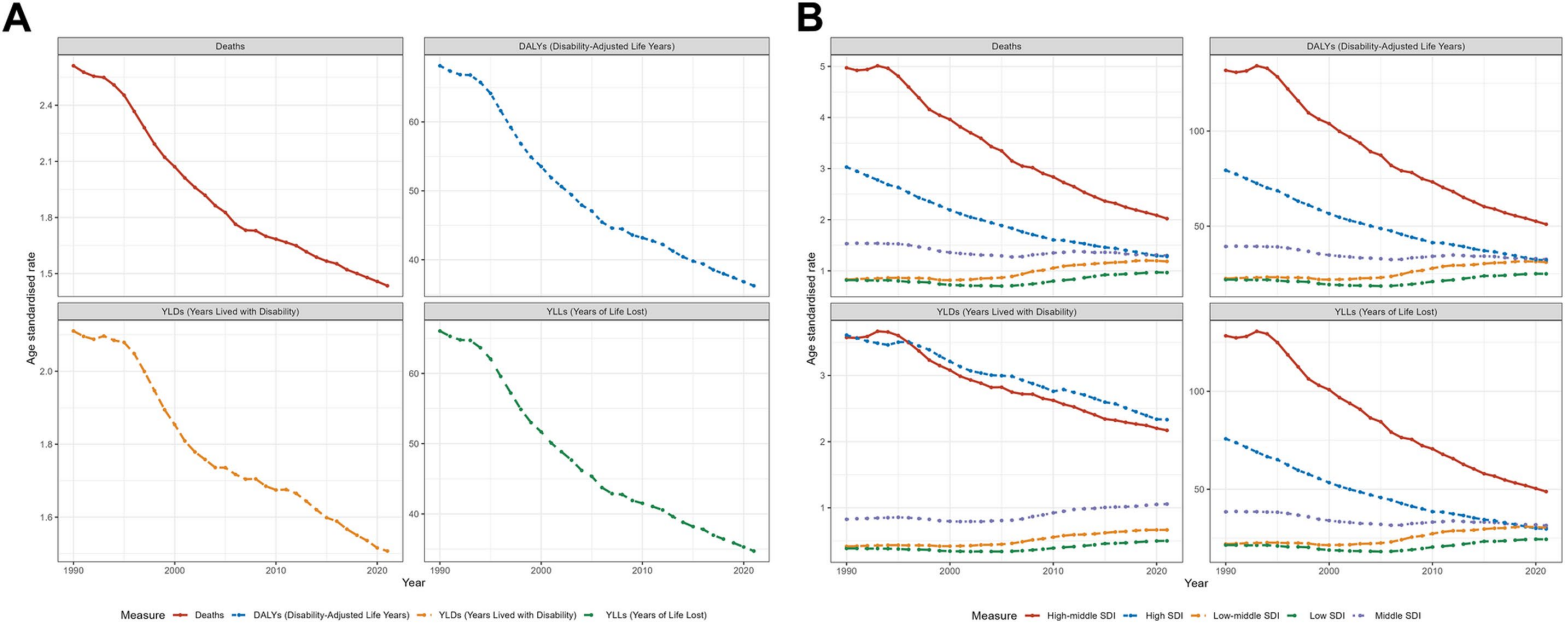
Trends in laryngeal cancer deaths, DALYs, YLDs, and YLLs from 1990 to 2021: **(A)** at the global level and **(B)** across different SDI levels, focusing on men over 50 years old and cases caused by alcohol use.

The burden of laryngeal cancer caused by alcohol showed a similar trend: increases in the cases of YLDs, deaths, YLLs, and DALYs, while the corresponding ASRs declined (Figure 2A). In 2021, high-middle SDI areas experienced the greatest burden in terms of ASRs for deaths, DALYs, and YLLs, while high SDI areas had the highest ASRs for YLDs (Figure 2B). The specific burden values, along with the trends in EAPC, are detailed in Supplementary Tables S3, S4.

### Health inequality trends

In 1990 and 2021, the SII values for laryngeal cancer DALYs linked to smoking were 221 and 23, respectively (Supplementary Figure S1A), while the concentration indices were 0.04 and −0.11 (Supplementary Figure S1B). This indicates a significant improvement in health inequality related to smoking-induced laryngeal cancer between countries of different income levels. However, the change in the concentration index suggests that inequality has shifted toward lower SDI regions.

For alcohol-induced laryngeal cancer DALYs, the SII values were 75 in 1990 and 21 in 2021 (Supplementary Figure S2A), with concentration indices of 0.28 and 0.06, respectively (Supplementary Figure S2B). These figures demonstrate significant improvements in health inequality related to alcohol-induced laryngeal cancer.

### Regional burden of laryngeal cancer from smoking and alcohol consumption

**Figure 3:**
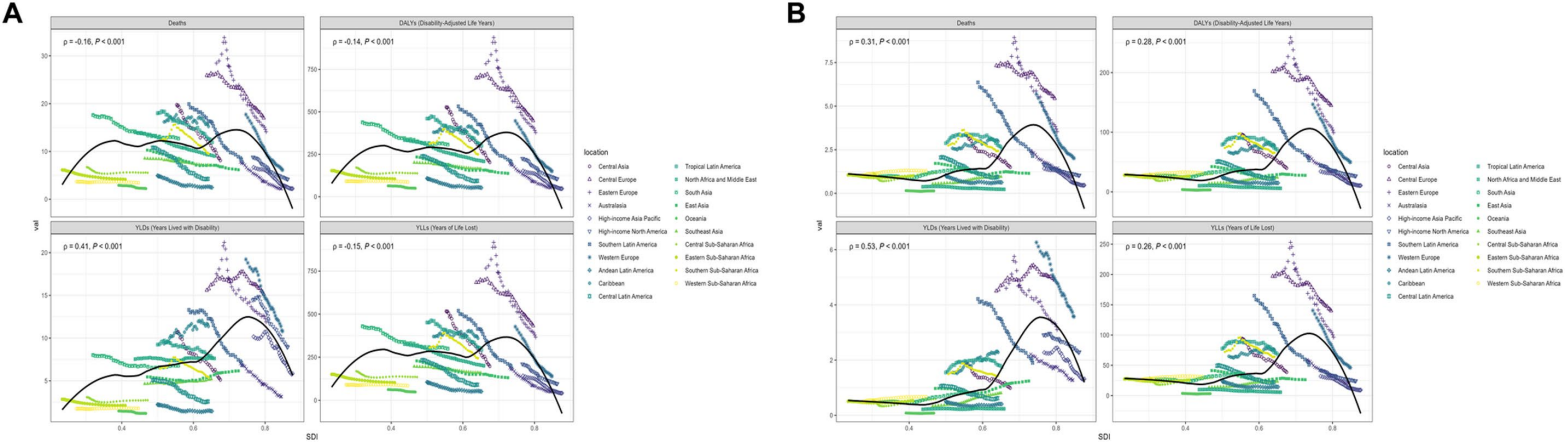
The trends in deaths, DALYs, YLDs, and YLLs for laryngeal cancer across 21 different regions and their relationship with SDI in **(A)** smoking-related laryngeal cancer and **(B)** alcohol-related laryngeal cancer.

The data for deaths, DALYs, YLLs, YLDs, and EAPC across various regions are presented in Supplementary Tables S1–S4. Graphs are provided to enhance data visualization. Figure 3 depicts the influence of SDI on the laryngeal cancer burden across 21 regions, with the X-axis representing SDI and the Y-axis corresponding to ASRs. A smoothed black curve shows the overall trend, while distinct icons represent the trends for each region.

### Key regional trends

Smoking-related laryngeal cancer (Figure 3A): The ASRs for deaths, DALYs, and YLLs initially increase, stabilize, and then decline as SDI rises. For YLDs, the overall trend is an increase followed by a decrease. The burden tends to decline more substantially in high SDI areas, whereas regions such as Central and Eastern Europe, the Caribbean, South Asia, and Tropical Latin America continue to experience comparatively higher burdens.

Alcohol-related laryngeal cancer (Figure 3B): The ASRs for deaths, DALYs, YLLs, and YLDs follow a stable-increasing-decreasing pattern with rising SDI. The burden has remained steady in middle- and low-SDI regions, whereas high-SDI areas have seen a notable reduction. Central and Eastern Europe bears the greatest burden in comparison.

### National burden of laryngeal cancer caused by smoking and alcohol use

**Figure 4:**
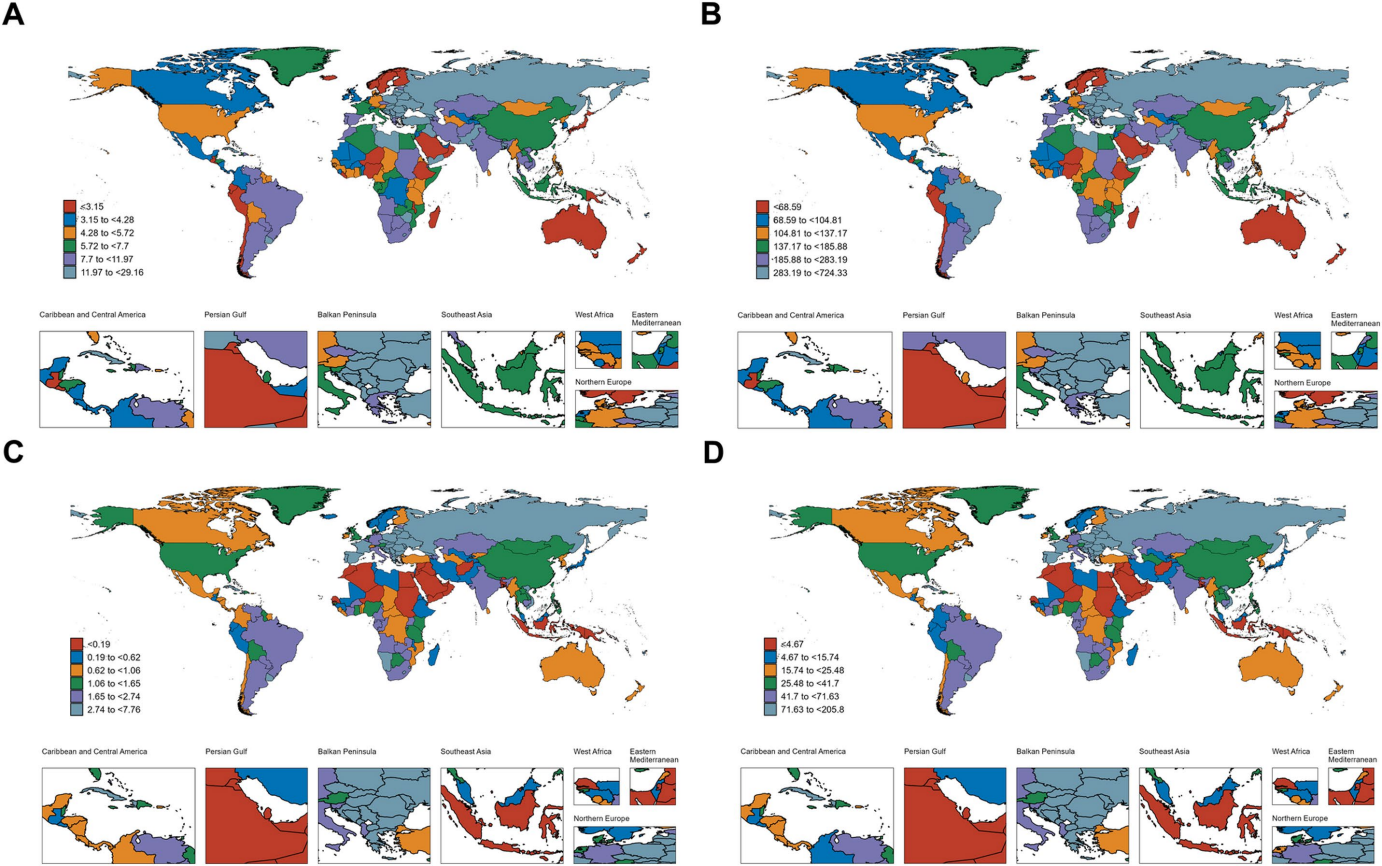
Geographical distribution of deaths and DALYs for **(A,B)** smoking-related laryngeal cancer; **(C,D)** alcohol-related laryngeal cancer.

Figures 4A,B visualize the burden of deaths and DALYs related to smoking-associated laryngeal cancer in various countries, while Figures 4C,D illustrate the burden for alcohol-associated cases. Supplementary Figures S3A,B demonstrate national burdens of YLDs and YLLs due to smoking, while Supplementary Figures S3C,D show YLDs and YLLs from alcohol-related cases.

In 2021, the three nations with the highest laryngeal cancer burden due to smoking, based on ASRs, were:

- Deaths: Cuba (29.16), Montenegro (28.61), Seychelles (27.98).
- DALYs: Montenegro (724.33), Cuba (717.68), Georgia (660.87).
- YLDs: Monaco (36.84), Montenegro (25.86), and Cuba (23.56).
- YLLs: Montenegro (698.48), Cuba (694.13), and Georgia (645.00).

For alcohol-related laryngeal cancer, the top three countries with the highest ASRs were:

- Deaths: Montenegro (7.76), Bulgaria (7.69), Romania (7.06).
- DALYs: Bulgaria (205.80), Montenegro (199.03), Romania (196.55).
- YLDs: Monaco (8.03), Montenegro (7.11), Romania (6.99).
- YLLs: Bulgaria (198.96), Montenegro (191.93), Romania (189.56).

### Decomposition analysis based on DALYs

**Figure 5:**
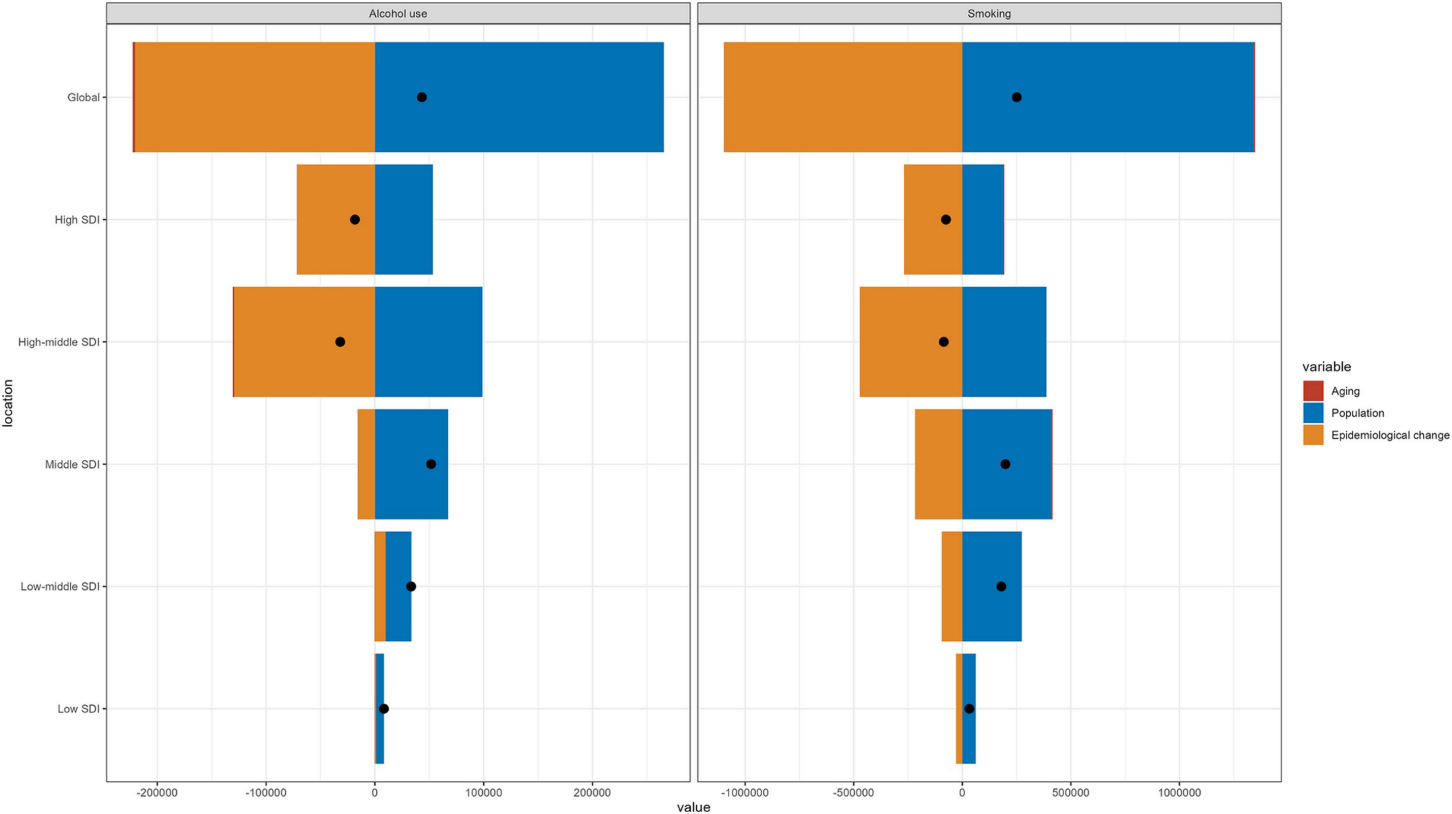
The decomposition analysis data for smoking- and alcohol-related laryngeal cancer at the global level and across different SDI regions.

Figure 5 illustrates the decomposition of DALYs by SDI level and globally:

Smoking-related laryngeal cancer: changes in DALYs were attributed 535.25% to population change, −437.81% to epidemiological change, and 2.55% to aging.

Alcohol-related laryngeal cancer: changes in DALYs were attributed 615.46% to population change, −510.68% to epidemiological change, and −4.78% to aging.

Overall, when other factors were held constant, population growth—the increase in the number of individuals aged 50 and above—played a dominant role in increasing DALYs associated with laryngeal cancer. In contrast, aging—the shift of the population into older age brackets beyond the 50-year threshold—had minimal impact on DALYs. Meanwhile, epidemiological changes—reflecting alterations in disease incidence, prevalence, mortality and others—had a negative impact on DALYs, second only in magnitude to the influence of population growth.

In high and high-middle SDI regions, the protective effects of epidemiological changes on DALYs outweighed the impact of population growth, indicating positive achievements in controlling the burden of laryngeal cancer in these areas. However, in middle, low-middle, and low SDI regions, the increase in DALYs driven by population growth was more pronounced, possibly reflecting higher fertility rates in these regions and indicating potential future challenges in managing the laryngeal cancer burden.

### Frontier analysis based on DALYs

**Figure 6:**
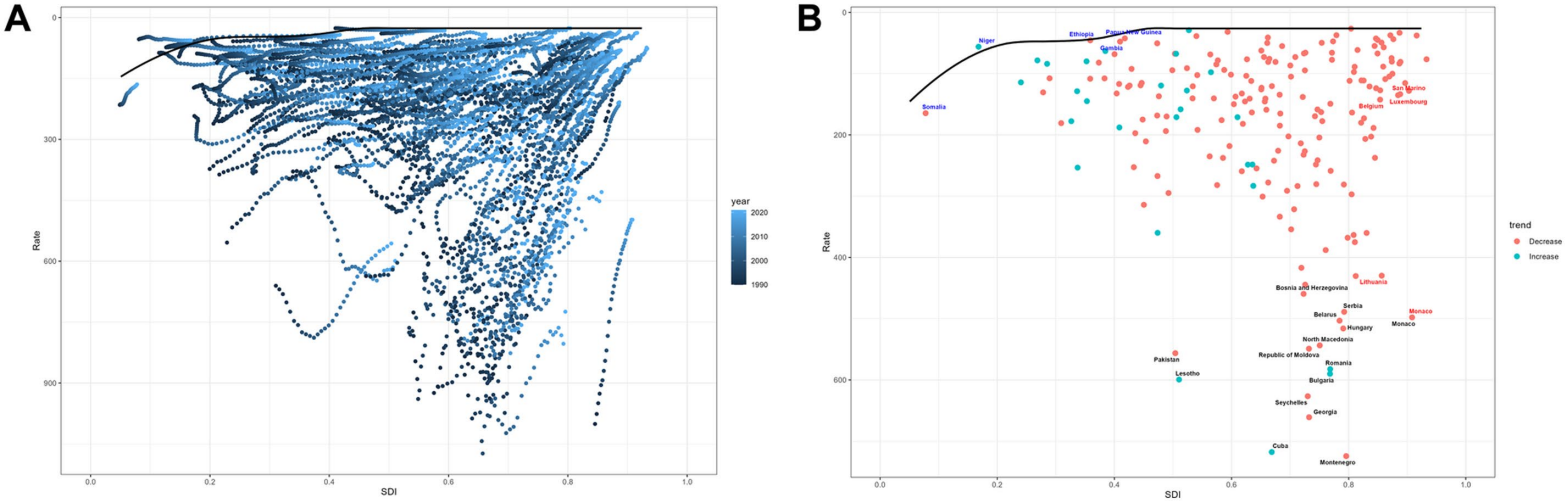
Frontier analysis on the basis of SDI and age-standardized DALYs of laryngeal cancer. **(A)** 1990–2021; **(B)** 2021.

Figure 6 presents the frontier analysis of DALYs (smoking-related laryngeal cancer) for 204 countries.

Figure 6A: shows that as SDI progresses, the burden follows a stable-fluctuating-declining trend.

Figure 6B: highlights 15 nations with the most significant disparities, shown in black font, with highest potential for reducing the burden: Pakistan, Lesotho, Cuba, Montenegro, Georgia, Seychelles, Bulgaria, Romania, Republic of Moldova, North Macedonia, Hungary, Belarus, Serbia, Bosnia and Herzegovina. Frontier nations with low-SDI (<0.5) include Somalia, Niger, Papua New Guinea, Ethiopia, and Gambia. Countries in high SDI (>0.85) regions with significant potential for improvement include Monaco, Lithuania, Belgium, Luxembourg, and San Marino.

### Projections of laryngeal cancer burden (2022–2040)

From 2022 to 2040, model predictions suggest increases in YLDs, DALYs, deaths, and YLLs for smoking- and alcohol-related laryngeal cancer, despite projected declines in ASRs.

Smoking-related projections by 2040:

- Deaths: 89,433
- DALYs: 2,076,123
- YLDs: 84,272
- YLLs: 1,992,685
- ASRs: 6.32, 150.56, 6.05, and 144.56, respectively.

Alcohol-related projections by 2040:

- Deaths: 16,291
- DALYs: 398,438
- YLDs: 17,588
- YLLs: 381,009
- ASRs: 1.17, 29.19, 1.28, and 27.93, respectively.

**Figure 7:**
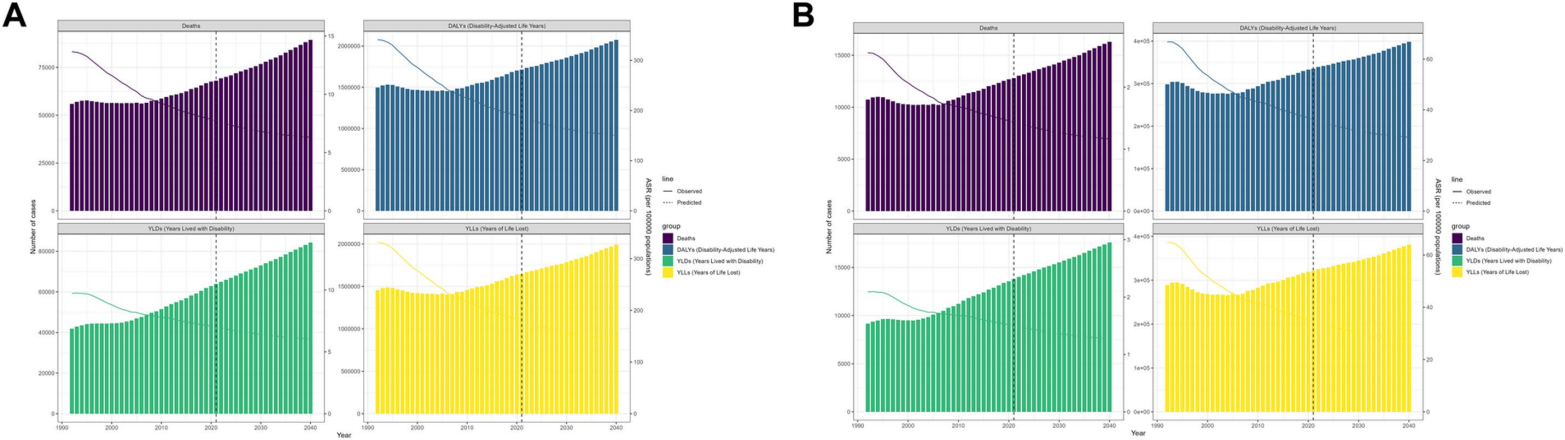
The age-standardized deaths, DALYs, YLDs, and YLLs for global laryngeal cancer during the observational period and the projection period in **(A)** smoking-related laryngeal cancer and **(B)** alcohol-related laryngeal cancer.

Figures 7A,B visualize the forecasted burden.

## Discussion

To our knowledge, this study is the first to analyze the reducible burden of laryngeal cancer at global, regional, and national levels based on its primary risk factors: smoking, alcohol use, advanced age, and male gender. Our study adds detailed information to the limited epidemiological data on high-risk laryngeal cancer patients.

Over the past years, various factors have been proven to be related to the development of laryngeal cancer, including but not limited to: smoking, alcohol consumption, HPV infection, gastroesophageal reflux, the role of pharyngeal microbiota, genetics, diet, and occupational exposure (6). Among these, smoking and alcohol are both major risk factors for laryngeal cancer, with evidence suggesting a synergistic effect in elevating cancer risk (2, 6, 21). While their individual contributions are well established, growing evidence indicates that these exposures may also interact with other carcinogenic factors. For instance, tobacco and alcohol may promote persistent HPV infection, increase the risk of gastroesophageal reflux, contribute to DNA damage and genetic mutations, and act synergistically with occupational exposures such as asbestos to increase cancer risk (22–26). Additionally, emerging evidence implicates microbiota dysbiosis in laryngeal cancer. Smoking is known to alter the composition of the upper aerodigestive tract microbiota, potentially influencing the metabolism of carcinogenic compounds and further increasing the risk of head and neck oncogenesis (27). Alcohol consumption may promote the enrichment of *Fusobacterium nucleatum*, which can disrupt ethanol metabolism, impair DNA mismatch repair, and enhance cellular proliferation, thereby potentially worsening the prognosis of head and neck cancers, including laryngeal cancer (28, 29). These complex and interacting risk pathways underscore the multifactorial nature of laryngeal cancer and highlight the importance of assessing disease burden within specific high-risk populations. Therefore, this study investigates the burden of laryngeal cancer attributable to smoking and alcohol use in individuals aged 50 years and older based on data from the GBD 2021.

From 1990 to 2021, the absolute burden of smoking- and alcohol-related laryngeal cancer in men over 50 increased, while the corresponding ASRs declined. This rise in absolute numbers largely reflects demographic changes—the growing and aging male population over 50, which expands the pool of individuals at risk. In contrast, the observed decline in ASRs may be attributed to the success of public health measures: since the implementation of the WHO Framework Convention on Tobacco Control, many signatory countries have introduced stricter tobacco control policies, contributing to a reduction in smoking prevalence in 116 countries (30). Improved diagnostic methods and accessibility to treatment have also enhanced the prognosis of laryngeal cancer (31). In addition, public awareness of the dangers of tobacco has continuously increased in some countries (32, 33), which may be an important contributing factor to the reduction in the burden of smoking-related diseases. Regarding alcohol consumption, the global per capita alcohol consumption has increased compared to 1990 (34), which seems to contradict the decreasing burden of ASRs for alcohol-related laryngeal cancer. However, some laryngeal cancer patients both smoke and drink, but smoking often plays the dominant role. Effective tobacco control may have reduced the combined carcinogenic impact of these behaviors.

In terms of the regional and national levels, Central Europe and Eastern Europe bear a higher burden, highlighting the regional heterogeneity of laryngeal cancer burden. Among the countries with the highest laryngeal cancer burden, European nations account for a significant proportion, including Montenegro, Georgia, Monaco, Bulgaria, and Romania. This may be associated with the high prevalence of smoking and alcohol consumption in Europe (35). In North America, Cuba also exhibits a high smoking rate (36). The high burden of laryngeal cancer in these regions and countries highlights the necessity of adjusting tobacco and alcohol policies. Beyond smoking and alcohol use, socioeconomic status, healthcare interventions, treatment accessibility, and patient outcomes may also play critical roles in explaining regional and national disparities in the burden of laryngeal cancer. Advanced-stage laryngeal cancer is associated with significantly higher mortality, highlighting the importance of early detection and screening in improving patient prognosis (37). However, in economically disadvantaged regions, access to routine screening remains limited (3). Patients with lower income or without health insurance are more likely to be diagnosed at later stages of disease (38, 39), which may restrict timely medical care and reduce opportunities for effective intervention.

When considering the burden of cancer patients, mortality rate is not the only criterion. DALYs, which integrate both mortality and disability-related losses, serve as a comprehensive indicator of laryngeal cancer burden. To further explore the influencing factors of DALYs in laryngeal cancer patients, we decomposed DALYs into three components—aging, population, and epidemiology. Among male laryngeal cancer patients over 50 years old, aging is no longer the primary factor affecting DALYs (2.55% for smoking and −4.78% for alcohol consumption). This is consistent with the conclusions from previous competing risks regression models, which suggest that as age reaches a certain threshold, other risk factors become more prominent (40). Population growth and epidemiological changes are the main contributors to the burden of laryngeal cancer in older adult males. Similar trends are observed across different SDI regions. With the continued growth of the global population, DALYs in this group may further increase. In response to these challenges, more adaptive and targeted policies are essential to effectively mitigate the growing burden of laryngeal cancer. Based on the risk factors identified, we recommend a multifaceted prevention strategy: strengthen tobacco and alcohol controls through higher taxes, advertising bans, and expanded cessation services; enforce workplace regulations to limit exposure to known carcinogens (e.g., asbestos, wood dust) and provide protective equipment; launch targeted health-education campaigns to raise awareness of laryngeal cancer symptoms and modifiable risks; promote HPV vaccination and effective management of gastroesophageal reflux; and support screening programs in high-risk populations. Tailoring these measures to local socio-economic and cultural contexts will maximize their impact on reducing the reducible burden of laryngeal cancer.

To assess the potential improvements in the burden of laryngeal cancer caused by smoking across different nations, a frontier analysis was performed based on DALYs. This analysis highlighted 15 nations with considerable potential for improving the burden of laryngeal cancer. In regions with an SDI below 0.5, Papua New Guinea, Ethiopia, and Gambia have successfully managed the burden of laryngeal cancer despite limited resources, serving as exemplary models. This achievement may be attributed to their active tobacco control efforts, such as strengthening tobacco taxation, banning tobacco advertising, establishing national strategic plans, and other measures (41–43). However, in some high-SDI countries, including Monaco, Lithuania, Belgium, Luxembourg, and San Marino, the burden of laryngeal cancer appears inconsistent with their level of development, warranting further reflection. These countries are all located in Europe, where historically high tobacco consumption is part of cultural norms (44). In some of these countries, the absence of comprehensive anti-smoking education and limited public awareness of tobacco-related harms may contribute to an increased disease burden (45). Additionally, in countries such as Monaco and San Marino, small population sizes may exaggerate ASRs, making the burden appear disproportionately high despite fewer absolute cases. The findings of the frontier analysis highlight the heterogeneity of disease across countries, highlighting the importance of tailored prevention strategies for each nation.

Using data from GBD 2021, we projected the future burden of laryngeal cancer in men aged 50 years and older through 2040 by applying the Nordpred age–period–cohort model, which extrapolates observed age-specific rates under a generalized linear model (46). The results indicate that the numbers of YLDs, DALYs, deaths, and YLLs due to laryngeal cancer will continue to rise, while the corresponding ASRs will further decline. The increase in the absolute number of laryngeal cancer cases can be attributed to the growth in the population of high-risk age groups. Therefore, better management of health issues related to laryngeal cancer is essential. Fortunately, the decrease in ASRs means a reduction in the average risk of the population. Although significant progress has been made in managing the burden of laryngeal cancer over the past three decades, there remains room for improvement in specific regions and countries. It is worth noting that these projections assume the continuation of current trends in risk-factor exposure, diagnostic practice, and treatment, and do not account for unexpected shifts—such as novel screening programs, policy changes, or emerging therapies—that could alter the trajectory. As such, our results should be interpreted as plausible scenarios rather than precise forecasts.

Prior to our study, a research investigation explored the global burden of laryngeal cancer linked to alcohol use and smoking (12). The findings indicated that, while the cases of laryngeal cancer due to smoking and drinking had increased globally, the corresponding ASRs had declined. This conclusion aligns with our findings. The strengths of that study lie in its subgroup analysis by gender and its in-depth examination of global, regional, and national trends using the EAPC. However, our study differs in several key aspects. First, building upon the risk factors of alcohol use and smoking, our research specifically focuses on the highest-risk population for laryngeal cancer: older male patients. We provide a detailed epidemiological characterization of this high-risk group and have updated the study data from 2019 to 2021. Second, leveraging the Nordpred model, we predict the future burden of laryngeal cancer in men over 50 years of age. Finally, through decomposition analysis and frontier analysis, we extensively explore the factors influencing DALYs related to laryngeal cancer and identify several countries with significant potential for burden reduction. Therefore, our study provides valuable insights for the management and mitigation of the global burden of laryngeal cancer.

Our study also has several limitations. First, the analysis relies heavily on estimates from the GBD 2021, which may limit the diversity of epidemiological data sources. As GBD burden estimates are generated through complex modeling techniques, they may deviate from actual disease patterns in certain regions, especially where local data are sparse or outdated. In the future, integrating GBD data with real-world epidemiological studies, national cancer registries, hospital-based surveillance systems, and cohort studies is expected to enhance the accuracy of disease burden estimates. Secondly, due to the scarcity of data, a more detailed analysis could not be conducted, such as the relationship between the location, staging of laryngeal cancer, and disease burden, in the future, a more detailed classification of cancer subsite and staging will be crucial for informing targeted health policies. Additionally, some risk factors may interact with smoking and drinking, and focusing solely on these two may further reduce data precision. Therefore, more clinical data are needed to support the interactions between smoking, alcohol use, and other risk factors in influencing laryngeal cancer risk. Such evidence would help improve the accuracy of the GBD model by refining its estimation of joint effects.

In conclusion, from 1990 to 2021, in males aged 50 and above, the absolute burden of smoking- and alcohol-related laryngeal cancer increased, while the corresponding ASRs have shown a declining trend, suggesting a lower average risk of laryngeal cancer in this population. By 2040, the disease burden is projected to continue increasing in absolute numbers, while ASRs will further decline. The implementation of tobacco control policies has been a key factor in the decline of ASRs. However, caution is needed as global alcohol consumption has risen compared to previous levels, which may become a detrimental factor for the laryngeal cancer burden. Marked differences in the burden of laryngeal cancer continue to exist between regions and nations, necessitating dynamic adjustments to tobacco and alcohol control policies in different areas.

## Data Availability

The original contributions presented in the study are included in the article/Supplementary material, further inquiries can be directed to the corresponding author.
