# Supplementary figures and images for: Reducible burden of laryngeal cancer in men aged 50 and older attributable to smoking and alcohol use: insights from the global burden of disease study 2021
Source: Front Public Health. 2025 Jun 9;13:1577138. doi: 10.3389/fpubh.2025.1577138 (PMC12183041; doi:10.3389/fpubh.2025.1577138)

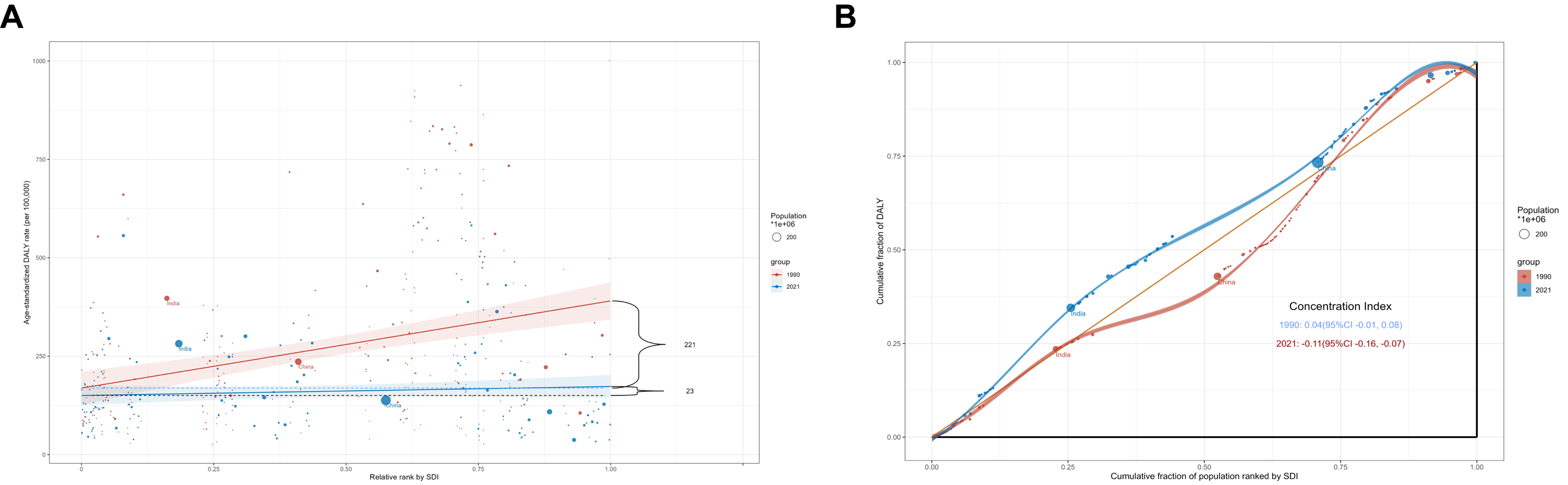

Supplement: Supplementary file 1 [file Image_1.TIF]

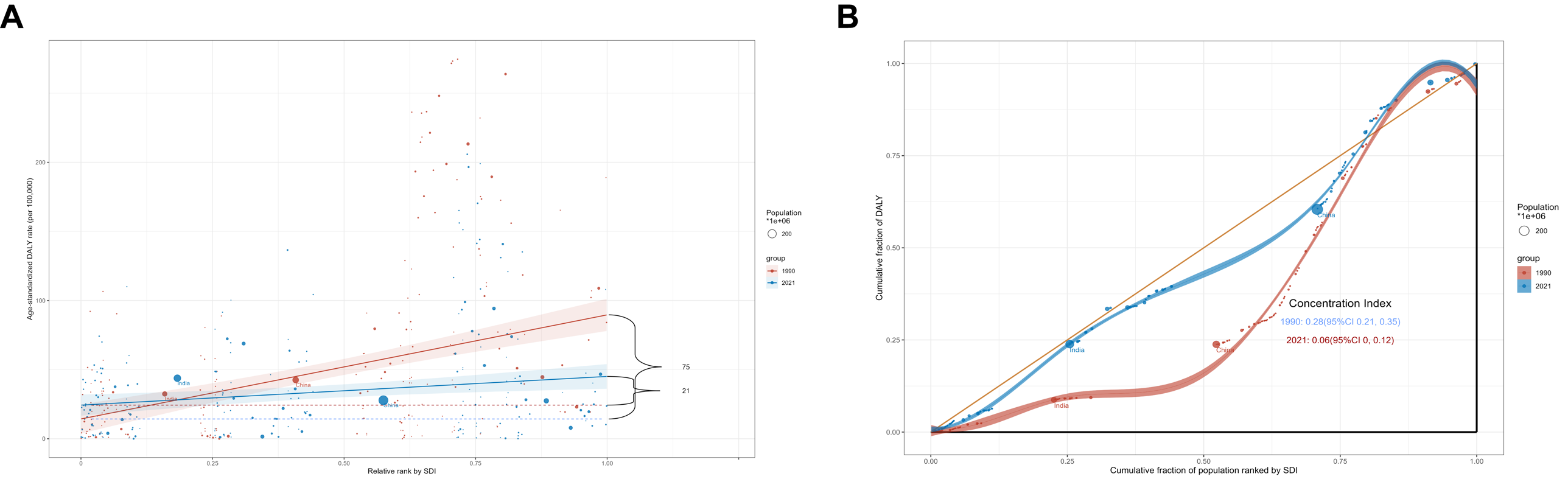

Supplement: Supplementary file 2 [file Image_2.TIF]

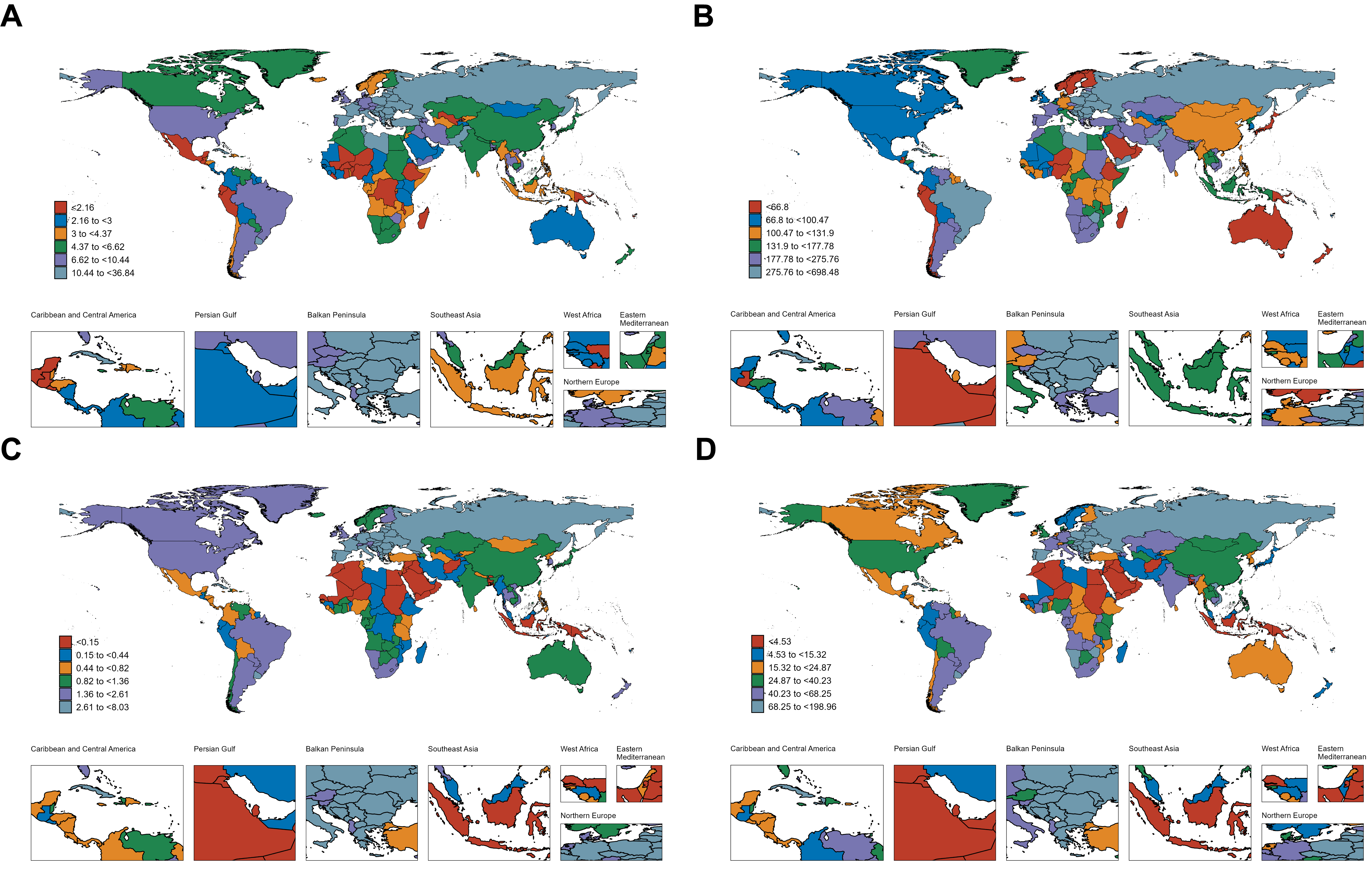

Supplement: Supplementary file 3 [file Image_3.TIF]
