# Supplementary material for: Reducible burden of laryngeal cancer in men aged 50 and older attributable to smoking and alcohol use: insights from the global burden of disease study 2021
Source: Front Public Health. 2025 Jun 9;13:1577138. doi: 10.3389/fpubh.2025.1577138 (PMC12183041; doi:10.3389/fpubh.2025.1577138)
Supplement: Supplementary file 4 [file Table_1.DOCX]

**Table S1.** Global deaths, DALYs, YLDs, and YLLs due to laryngeal cancer caused by smoking in men aged 50 and above in 2021.

| Location | Deaths (95% UI) | | DALYs (95% UI) | | YLDs (95% UI) | | YLLs (95% UI) | |
| --- | --- | --- | --- | --- | --- | --- | --- | --- |
|  | Numbers | ASRs | Numbers | ASRs | Numbers | ASRs | Numbers | ASRs |
| Global | 68063.16 (60152.35-76152.98) | 7.72 (6.80-8.65) | 1712955.35 (1523419.76-1917229.65) | 186.62 (165.72-208.98) | 63936.95 (46096.07-85343.95) | 7.04 (5.08-9.38) | 1649018.40 (1464404.13-1842744.46) | 179.58 (159.24-200.79) |
| High SDI | 9517.71 (8359.60-10600.24) | 4.51 (3.97-5.02) | 226068.57 (202057.52-249944.87) | 110.43 (98.89-121.91) | 16875.20 (12064.27-22638.30) | 8.19 (5.85-11.00) | 209193.37 (186910.04-230434.85) | 102.24 (91.54-112.45) |
| High-middle SDI | 17528.91 (15272.79-19971.03) | 8.80 (7.64-10.05) | 442528.08 (387674.16-502464.97) | 213.40 (186.62-242.56) | 18779.47 (13353.16-25409.39) | 9.09 (6.47-12.30) | 423748.61 (371403.66-480971.15) | 204.31 (178.72-232.15) |
| Middle SDI | 20556.17 (17471.70-23918.00) | 7.55 (6.40-8.79) | 515019.38 (438707.87-599462.33) | 175.57 (149.33-204.36) | 16876.13 (11815.78-23070.76) | 5.82 (4.08-7.94) | 498143.25 (424644.87-579552.93) | 169.75 (144.48-197.48) |
| Low-middle SDI | 16713.04 (14070.50-19752.04) | 11.53 (9.70-13.64) | 431122.58 (363068.70-510529.74) | 277.86 (233.97-329.11) | 9412.31 (6560.35-12966.09) | 6.18 (4.31-8.49) | 421710.27 (355031.80-498289.40) | 271.69 (228.68-321.11) |
| Low SDI | 3650.20 (2875.58-4560.82) | 7.28 (5.72-9.10) | 95743.46 (75396.76-119489.06) | 174.24 (137.10-217.59) | 1908.82 (1262.65-2748.04) | 3.58 (2.37-5.15) | 93834.64 (73969.17-117128.61) | 170.66 (134.42-213.15) |
| Andean Latin America | 145.36 (101.10-202.79) | 2.48 (1.72-3.46) | 3203.30 (2243.20-4461.00) | 52.79 (36.92-73.51) | 87.51 (52.51-136.14) | 1.45 (0.87-2.26) | 3115.78 (2181.96-4344.99) | 51.33 (35.90-71.58) |
| Australasia | 124.07 (90.56-163.88) | 2.22 (1.63-2.93) | 2838.79 (2109.30-3665.72) | 53.08 (39.48-68.42) | 167.26 (103.15-257.65) | 3.16 (1.94-4.89) | 2671.53 (1985.43-3458.29) | 49.92 (37.14-64.47) |
| Caribbean | 844.50 (652.44-1064.10) | 15.24 (11.76-19.21) | 20989.03 (16281.18-26356.05) | 371.02 (287.71-465.91) | 641.24 (398.27-965.03) | 11.38 (7.08-17.11) | 20347.79 (15781.16-25571.11) | 359.64 (278.84-451.98) |
| Central Asia | 637.08 (555.41-724.72) | 8.10 (7.05-9.23) | 17491.81 (15235.97-19920.51) | 202.43 (176.19-230.62) | 433.05 (304.01-600.09) | 5.12 (3.59-7.08) | 17058.77 (14878.83-19404.04) | 197.31 (171.93-224.53) |
| Central Europe | 3579.19 (3125.94-4029.66) | 16.99 (14.82-19.15) | 92916.17 (81420.87-104287.22) | 447.84 (392.30-502.87) | 3137.67 (2248.03-4250.19) | 15.15 (10.85-20.53) | 89778.50 (78803.73-100761.31) | 432.69 (379.63-485.87) |
| Central Latin America | 1030.65 (827.51-1253.78) | 4.26 (3.42-5.18) | 23492.30 (18923.84-28699.16) | 93.20 (75.04-113.81) | 645.26 (433.09-903.88) | 2.58 (1.73-3.61) | 22847.05 (18391.51-27838.30) | 90.62 (72.92-110.36) |
| Central Sub-Saharan Africa | 285.60 (189.75-414.09) | 5.54 (3.69-8.03) | 8140.68 (5376.51-11862.28) | 137.44 (91.48-199.33) | 152.72 (87.82-246.41) | 2.73 (1.58-4.40) | 7987.96 (5271.92-11633.47) | 134.71 (89.59-195.28) |
| East Asia | 13861.79 (10222.49-18104.90) | 6.18 (4.59-8.00) | 334505.94 (244522.82-440574.86) | 137.30 (100.91-179.98) | 15014.89 (9513.40-22147.54) | 6.17 (3.93-9.06) | 319491.05 (233229.62-421479.02) | 131.13 (96.24-172.19) |
| Eastern Europe | 4373.47 (3761.50-4999.88) | 14.13 (12.14-16.17) | 119605.57 (102720.42-136936.81) | 378.07 (324.37-433.09) | 3753.87 (2631.39-5038.38) | 11.91 (8.35-16.01) | 115851.71 (99589.69-132698.35) | 366.16 (314.41-419.65) |
| Eastern Sub-Saharan Africa | 689.29 (505.94-918.31) | 4.15 (3.05-5.49) | 18999.08 (13887.79-25561.62) | 104.17 (76.38-139.08) | 370.65 (231.64-571.00) | 2.12 (1.33-3.23) | 18628.43 (13619.31-25057.86) | 102.06 (74.85-136.24) |
| High-income Asia Pacific | 1107.38 (912.03-1307.54) | 2.25 (1.85-2.66) | 21617.96 (17951.30-25740.56) | 47.59 (39.50-56.98) | 2608.36 (1768.49-3636.07) | 5.85 (3.96-8.19) | 19009.59 (15778.64-22525.85) | 41.74 (34.61-49.73) |
| High-income North America | 2839.65 (2461.70-3193.63) | 4.22 (3.65-4.75) | 69984.23 (61754.00-77730.98) | 105.15 (92.81-116.79) | 6004.41 (4253.59-8162.56) | 8.98 (6.36-12.20) | 63979.82 (56572.31-70965.12) | 96.17 (85.06-106.68) |
| North Africa and Middle East | 4162.71 (3416.79-5038.13) | 9.03 (7.40-10.95) | 105623.17 (86973.48-127531.61) | 208.20 (171.19-251.50) | 3804.27 (2560.55-5363.20) | 7.63 (5.13-10.74) | 101818.90 (83761.77-123185.11) | 200.57 (164.78-242.67) |
| Oceania | 15.98 (10.76-23.39) | 2.24 (1.51-3.27) | 408.14 (273.13-602.29) | 49.40 (33.19-72.51) | 9.37 (5.72-15.19) | 1.19 (0.73-1.92) | 398.77 (267.10-587.63) | 48.21 (32.42-70.63) |
| South Asia | 19418.92 (15900.03-23338.96) | 12.72 (10.42-15.28) | 499434.78 (407962.46-602486.80) | 306.45 (250.52-369.42) | 10884.37 (7421.92-15115.38) | 6.78 (4.63-9.39) | 488550.41 (399182.05-588663.11) | 299.67 (245.03-360.84) |
| Southeast Asia | 4441.16 (3607.18-5425.67) | 7.10 (5.75-8.69) | 114482.89 (92800.74-140292.32) | 164.41 (133.27-201.29) | 3553.17 (2390.16-5067.30) | 5.20 (3.49-7.41) | 110929.72 (90046.77-135768.12) | 159.21 (129.19-194.73) |
| Southern Latin America | 660.02 (522.25-818.98) | 7.76 (6.12-9.66) | 16422.12 (13211.35-20180.74) | 192.02 (154.31-236.22) | 584.16 (357.53-882.49) | 6.83 (4.18-10.33) | 15837.97 (12740.07-19424.65) | 185.19 (148.79-227.35) |
| Southern Sub-Saharan Africa | 503.95 (409.38-603.89) | 9.56 (7.73-11.49） | 14282.51 (11617.56-17126.42) | 249.19 (202.30-298.89) | 304.71 (205.42-423.56) | 5.45 (3.66-7.59) | 13977.80 (11372.99-16762.86) | 243.74 (197.94-292.38) |
| Tropical Latin America | 3030.61 (2566.64-3496.11) | 11.90 (10.04-13.77) | 79201.76 (67517.06-90842.79) | 299.41 (254.66-343.93) | 2023.84 (1394.41-2769.19) | 7.70 (5.29-10.54) | 77177.92 (65852.68-88529.49) | 291.71 (248.35-335.15) |
| Western Europe | 5640.55 (4858.44-6462.92) | 6.14 (5.32-7.01) | 130923.40 (114560.51-148234.31) | 150.57 (132.23-170.04) | 9389.78 (6530.75-12723.88) | 10.81 (7.52-14.66) | 121533.62 (106487.76-137656.28) | 139.76 (122.95-157.86) |
| Western Sub-Saharan Africa | 671.21 (494.90-858.02) | 3.45 (2.55-4.43) | 18391.72 (13510.55-23435.20) | 86.66 (63.87-110.63) | 366.39 (235.74-534.56) | 1.79 (1.15-2.60) | 18025.33 (13238.86-22971.96) | 84.88 (62.53-108.38) |

DALYs, disability-adjusted life years; YLDs, years lived with disability; YLLs, years of life lost; UI, uncertainty intervals; ASRs, age-standardized rates; SDI, socio‑demographic index.

**Table S2.** EAPC of laryngeal cancer due to smoking in men aged 50 and above from 1990 to 2021.

| Location | EAPC: deaths (95% UI) | EAPC: DALYs (95% UI) | EAPC: YLDs (95% UI) | EAPC: YLLs (95% UI) |
| --- | --- | --- | --- | --- |
| Global | -2.05 (-2.12 to -1.98) | -2.17 (-2.24 to -2.09) | -1.18 (-1.24 to -1.11) | -2.20 (-2.28 to -2.12) |
| High SDI | -3.09 (-3.17 to -3.01) | -3.15 (-3.22 to -3.09) | -1.71 (-1.79 to -1.64) | -3.25 (-3.32 to -3.18) |
| High-middle SDI | -2.90 (-2.99 to -2.81) | -3.10 (-3.20 to -3.00) | -1.45 (-1.53 to -1.36) | -3.16 (-3.26 to -3.05) |
| Middle SDI | -1.53 (-1.59 to -1.48) | -1.61 (-1.67 to -1.55) | -0.17 (-0.27 to -0.08) | -1.65 (-1.70 to -1.59) |
| Low-middle SDI | -0.75 (-0.81 to -0.69) | -0.84 (-0.90 to -0.78) | -0.28 (-0.37 to -0.19) | -0.85 (-0.91 to -0.79) |
| Low SDI | -1.02 (-1.10 to -0.95) | -1.18 (-1.26 to -1.10) | -0.75 (-0.83 to -0.68) | -1.19 (-1.27 to -1.11) |
| Andean Latin America | -2.44 (-2.66 to -2.21) | -2.52 (-2.77 to -2.28) | -1.63 (-1.89 to -1.36) | -2.55 (-2.79 to -2.31) |
| Australasia | -4.15 (-4.25 to -4.05) | -4.25 (-4.36 to -4.14) | -2.56 (-2.66 to -2.45) | -4.34 (-4.45 to -4.23) |
| Caribbean | -0.15 (-0.27 to -0.03) | 0.03 (-0.08 to 0.15) | 0.71 (0.59 to 0.84) | 0.02 (-0.10 to 0.13) |
| Central Asia | -2.81 (-2.95 to -2.67) | -3.08 (-3.22 to -2.94) | -2.32 (-2.42 to -2.23) | -3.10 (-3.24 to -2.96) |
| Central Europe | -1.48 (-1.59 to -1.37) | -1.58 (-1.70 to -1.45) | -0.16 (-0.30 to -0.01) | -1.62 (-1.74 to -1.49) |
| Central Latin America | -3.43 (-3.59 to -3.26) | -3.48 (-3.65 to -3.31) | -2.64 (-2.83 to -2.46) | -3.50 (-3.67 to -3.33) |
| Central Sub-Saharan Africa | -0.60 (-0.77 to -0.44) | -0.70 (-0.87 to -0.54) | -0.39 (-0.58 to -0.19) | -0.71 (-0.87 to -0.55) |
| East Asia | -1.63 (-1.74 to -1.52) | -1.71(-1.84 to -1.58) | 0.57 (0.38 to 0.76) | -1.79(-1.92 to -1.66) |
| Eastern Europe | -2.83 (-3.05 to -2.60) | -2.97 (-3.20 to -2.73) | -1.71 (-1.89 to -1.52) | -3.00 (-3.23 to -2.77) |
| Eastern Sub-Saharan Africa | -1.45 (-1.52 to -1.38) | -1.45 (-1.51 to -1.38) | -1.15 (-1.23 to -1.08) | -1.45 (-1.51 to -1.39) |
| High-income Asia Pacific | -4.01 (-4.20 to -3.82) | -4.18 (-4.35 to -4.02) | -2.04 (-2.26 to -1.82) | -4.41 (-4.58 to -4.23) |
| High-income North America | -2.62 (-2.68 to -2.55) | -2.68 (-2.75 to -2.62) | -1.80 (-1.91 to -1.68) | -2.76 (-2.82 to -2.69) |
| North Africa and Middle East | -1.48 (-1.54 to -1.43) | -1.60 (-1.65 to -1.55) | 0.02 (-0.04 to 0.07) | -1.65 (-1.70 to -1.59) |
| Oceania | -0.97 (-1.04 to -0.90) | -0.95 (-1.02 to -0.89) | -0.84 (-0.91 to -0.76) | -0.95 (-1.02 to -0.89) |
| South Asia | -1.19 (-1.28 to -1.11) | -1.31 (-1.39 to -1.22) | -0.69 (-0.81 to -0.58) | -1.32(-1.41 to -1.23) |
| Southeast Asia | -0.67 (-0.70 to -0.64) | -0.70 (-0.73 to -0.68) | 0.32 (0.28 to 0.36) | -0.73 (-0.76 to -0.71) |
| Southern Latin America | -3.02 (-3.20 to -2.84) | -3.34 (-3.52 to -3.15) | -2.30 (-2.50 to -2.10) | -3.37 (-3.56 to -3.18) |
| Southern Sub-Saharan Africa | -1.10 (-1.45 to -0.74) | -1.00 (-1.37 to -0.63) | -0.78 (-1.03 to -0.53) | -1.00 (-1.38 to -0.63) |
| Tropical Latin America | -1.33 (-1.43 to -1.23) | -1.42 (-1.52 to -1.31) | -0.64 (-0.72 to -0.55) | -1.44 (-1.54 to -1.33) |
| Western Europe | -3.42 (-3.52 to -3.31) | -3.48 (-3.57 to -3.38) | -1.81 (-1.88 to -1.73) | -3.58 (-3.68 to -3.48) |
| Western Sub-Saharan Africa | -0.07 (-0.14 to -0.01) | -0.05 (-0.12 to 0.01) | 0.18 (0.13 to 0.24) | -0.06 (-0.13 to 0.01) |

EAPC, estimated average percentage change; UI, uncertainty intervals; DALYs, disability-adjusted life years; YLDs, years lived with disability; YLLs, years of life lost;

SDI, socio‑demographic index.

**Table S3.** Global deaths, DALYs, YLDs, and YLLs due to laryngeal cancer caused by drinking in men aged 50 and above in 2021.

| Location | Deaths (95% UI) | | DALYs (95% UI) | | YLDs (95% UI) | | YLLs (95% UI) | |
| --- | --- | --- | --- | --- | --- | --- | --- | --- |
|  | Numbers | ASRs | Numbers | ASRs | Numbers | ASRs | Numbers | ASRs |
| Global | 12785.54 (6825.74-18389.16) | 1.43 (0.76-2.07) | 335033.08 (179884.78-479265.05) | 36.22 (19.42-51.88) | 13800.14 (7030.82-21610.20) | 1.51 (0.77-2.36) | 321232.94 (172378.20-460004.20) | 34.72 (18.60-49.78) |
| High SDI | 2686.39 (1449.94-3797.10) | 1.28 (0.69-1.81) | 65340.82 (35553.63-91385.45) | 32.11 (17.52-44.85) | 4771.41 (2410.49-7454.22) | 2.33 (1.18-3.64) | 60569.40 (33041.89-84826.18) | 29.78 (16.29-41.65) |
| High-middle SDI | 4069.57 (2218.88-5830.18) | 2.02 (1.10-2.90) | 106508.93 (58381.56-151624.42) | 51.00 (27.90-72.68) | 4517.37 (2337.81-7072.35) | 2.17 (1.12-3.40) | 101991.56 (55926.16-145426.35) | 48.83 (26.72-69.70) |
| Middle SDI | 3686.25 (1920.02-5432.81) | 1.30 (0.67-1.92) | 98371.66 (51623.77-144095.34) | 32.60 (17.04-47.84) | 3155.32 (1568.33-5107.25) | 1.06 (0.52-1.71) | 95216.34 (49927.43-139651.34) | 31.55 (16.48-46.35) |
| Low-middle SDI | 1804.09 (854.46-2831.34) | 1.18 (0.56-1.86) | 49862.01 (23728.93-77772.24) | 31.09 (14.76-48.59) | 1053.87 (472.34-1807.94) | 0.67 (0.30-1.14) | 48808.15 (23216.44-76229.91) | 30.42 (14.44-47.61) |
| Low SDI | 514.11 (243.98-822.80) | 0.97 (0.45-1.55) | 14292.47 (6858.10-22728.69) | 24.97 (11.89-) | 279.26 (119.79-486.42) | 0.50 (0.21-0.87) | 14013.22 (6724.71-22308.21) | 24.47 (11.65-39.07) |
| Andean Latin America | 37.63 (16.18-63.19) | 0.63 (0.27-1.05) | 912.17 (400.83-1522.08) | 14.76 (6.46-24.65) | 24.89 (10.07-45.21) | 0.41 (0.16-0.74) | 887.28 (389.92-1481.23) | 14.36 (6.28-23.99) |
| Australasia | 52.69 (26.66-79.87) | 0.94 (0.48-1.43) | 1175.93 (614.76-1753.57) | 21.93 (11.54-32.62) | 67.81 (32.16-115.63) | 1.28 (0.61-2.19) | 1108.11 (579.08-1652.50) | 20.65 (10.86-30.72) |
| Caribbean | 167.12 (78.10-262.59) | 2.98 (1.39-4.70) | 4419.46 (2100.75-6877.65) | 77.48 (36.76-120.76) | 129.64 (55.99-225.95) | 2.28 (0.98-3.98) | 4289.82 (2035.92-6676.69) | 75.19 (35.62-117.21) |
| Central Asia | 124.73 (57.86-190.54) | 1.50 (0.68-2.33) | 3569.00 (1684.73-5384.97) | 39.65 (18.38-60.50) | 87.47 (38.29-145.39) | 0.99 (0.42-1.66) | 3481.53 (1644.66-5258.84) | 38.66 (17.94-59.04) |
| Central Europe | 1143.71 (636.58-1618.84) | 5.44 (3.03-7.70) | 29975.00 (16971.40-42138.15) | 144.92 (82.24-203.63) | 1012.00 (532.65-1572.92) | 4.90 (2.57-7.62) | 28963.00 (16394.86-40733.87) | 140.02 (79.41-196.77) |
| Central Latin America | 235.28 (108.09-365.22) | 0.94 (0.43-1.47) | 5906.96 (2765.96-9097.70) | 22.86 (10.65-35.28) | 159.05 (70.87-267.82) | 0.62 (0.28-1.05) | 5747.91 (2691.36-8840.15) | 22.24 (10.36-34.27) |
| Central Sub-Saharan Africa | 62.54 (24.30-110.13) | 1.18 (0.45-2.10) | 1806.85 (703.24-3179.79) | 29.92 (11.58-52.80) | 33.93 (12.70-65.19) | 0.59 (0.22-1.14) | 1772.92 (690.50-3120.53) | 29.34 (11.36-51.74) |
| East Asia | 2678.93 (1329.98-4178.01) | 1.15 (0.57-1.79) | 68745.19 (34476.76-107499.28) | 27.53 (13.77-43.05) | 3090.89 (1464.42-5247.78) | 1.24 (0.59-2.11) | 65654.30 (32930.37-102363.27) | 26.29 (13.15-41.00) |
| Eastern Europe | 1115.04 (533.31-1677.27) | 3.54 (1.68-5.37) | 31681.14 (15615.00-46989.04) | 99.42 (48.87-148.10) | 986.93 (450.45-1592.58) | 3.10 (1.41-5.03) | 30694.21 (15143.44-45495.90) | 96.32 (47.40-143.36) |
| Eastern Sub-Saharan Africa | 172.67 (82.55-279.80) | 1.00 (0.48-1.62) | 4903.37 (2342.69-8000.85) | 26.20 (12.51-42.52) | 95.98 (41.94-172.00) | 0.53 (0.23-0.94) | 4807.40 (2298.26-7841.52) | 25.67 (12.27-41.66) |
| High-income Asia Pacific | 224.23 (113.22-340.08) | 0.47 (0.24-0.71) | 4654.56 (2390.30-7015.03) | 10.56 (5.48-15.90) | 570.83 (272.21-954.23) | 1.32 (0.63-2.21) | 4083.73 (2115.90-6168.51) | 9.24 (4.84-13.95) |
| High-income North America | 698.79 (325.79-1044.18) | 1.04 (0.49-1.56) | 17680.08 (8402.47-26042.55) | 26.71 (12.74-39.33) | 1529.13 (698.28-2474.43) | 2.30 (1.05-3.72) | 16150.95 (7709.37-23823.06) | 24.41 (11.69-35.99) |
| North Africa and Middle East | 114.63 (44.70-200.97) | 0.23 (0.09-0.41) | 3165.22 (1248.23-5525.74) | 5.92 (2.32-10.36) | 124.13 (44.84-236.62) | 0.23 (0.08-0.45) | 3041.09 (1201.29-5315.06) | 5.69 (2.23-9.96) |
| Oceania | 1.06 (0.38-1.97) | 0.14 (0.05-0.26) | 28.26 (10.22-52.82) | 3.25 (1.16-6.09) | 0.66 (0.22-1.30) | 0.08 (0.03-0.16) | 27.6 (9.97-51.59) | 3.17 (1.14-5.94) |
| South Asia | 2258.31 (1025.58-3722.34) | 1.41 (0.64-2.33) | 62368.11 (28396.12-102321.71) | 37.11 (16.85-60.98) | 1370.69 (596.74-2415.96) | 0.82 (0.36-1.45) | 60997.42 (27784.04-100092.06) | 36.28 (16.49-59.64) |
| Southeast Asia | 633.04 (321.11-965.34) | 0.95 (0.48-1.46) | 17407.42 (8889.67-26502.24) | 23.88 (12.13-36.44) | 578.45 (278.96-1006.88) | 0.80 (0.38-1.40) | 16828.96 (8584.95-25582.87) | 23.07 (11.72-35.11) |
| Southern Latin America | 181.54 (91.70-272.06) | 2.13 (1.08-3.21) | 4499.54 (2296.63-6681.98) | 52.66 (26.85-78.22) | 162.25 (70.77-281.33) | 1.90 (0.83-3.29) | 4337.29 (2214.88-6458.19) | 50.76 (25.90-75.60) |
| Southern Sub-Saharan Africa | 131.60 (65.50-196.60) | 2.43 (1.20-3.64) | 3817.81 (1906.98-5695.75) | 65.52 (32.61-97.85) | 81.82 (39.46-134.01) | 1.44 (0.69-2.36) | 3735.99 (1867.05-5578.31) | 64.08 (31.91-95.78) |
| Tropical Latin America | 666.99 (340.00-994.75) | 2.56 (1.30-3.84) | 18479.15 (9505.12-27295.73) | 68.86 (35.33-101.97) | 466.68 (218.79-764.43) | 1.75 (0.82-2.87) | 18012.47 (9282.42-26584.14) | 67.11 (34.49-99.30) |
| Western Europe | 1837.96 (1005.38-2592.95) | 2.01 (1.11-2.83) | 43103.33 (23978.64-60227.12) | 49.80 (27.85-69.40) | 3090.17 (1603.63-4814.35) | 3.57 (1.86-5.56) | 40013.16 (22296.08-55899.65) | 46.23 (25.91-64.39) |
| Western Sub-Saharan Africa | 247.04 (114.67-377.92) | 1.28 (0.60-1.97) | 6734.52 (3123.46-10289.29) | 31.80 (14.75-48.62) | 136.74 (63.31-232.47) | 0.67 (0.31-1.13) | 6597.78 (3060.29-10078.92) | 31.13 (14.44-47.60) |

DALYs, disability-adjusted life years; YLDs, years lived with disability; YLLs, years of life lost; UI, uncertainty intervals; ASRs, age-standardized rates; SDI, socio‑demographic index.

**Table S4.** EAPC of laryngeal cancer due to drinking in men aged 50 and above from 1990 to 2021.

| Location | EAPC: deaths (95% UI) | EAPC: DALYs (95% UI) | EAPC: YLDs (95% UI) | EAPC: YLLs (95% UI) |
| --- | --- | --- | --- | --- |
| Global | -2.03 (-2.15 to -1.91) | -2.15 (-2.27 to -2.03) | -1.14 (-1.22 to -1.06) | -2.18 (-2.31 to -2.06) |
| High SDI | -2.81 (-2.90 to -2.72) | -2.93 (-3.01 to -2.85) | -1.43 (-1.49 to -1.36) | -3.02 (-3.11 to -2.94) |
| High-middle SDI | -3.16 (-3.25 to -3.06) | -3.34 (-3.44 to -3.24) | -1.79 (-1.88 to -1.71) | -3.39 (-3.50 to -3.29) |
| Middle SDI | -0.50 (-0.66 to -0.34) | -0.59 (-0.74 to -0.43) | 0.89 (0.67 to 1.11) | -0.63 (-0.77 to -0.48) |
| Low-middle SDI | 1.46 (1.24 to 1.69) | 1.35 (1.12 to 1.58) | 1.81 (1.57 to 2.05) | 1.34 (1.12 to 1.57) |
| Low SDI | 0.67 (0.34 to 1.00) | 0.51 (0.20 to 0.83) | 0.96 (0.62 to 1.31) | 0.51 (0.19 to 0.82) |
| Andean Latin America | -1.55 (-1.91 to -1.20) | -1.68 (-2.03 to -1.32) | -0.62 (-1.00 to -0.24) | -1.70 (-2.05 to -1.35) |
| Australasia | -3.01 (-3.13 to -2.88) | -3.27 (-3.39 to -3.14) | -1.63 (-1.78 to -1.48) | -3.35 (-3.47 to -3.22) |
| Caribbean | 0.69 (0.58 to 0.81) | 0.76 (0.64 to 0.88) | 1.50 (1.36 to 1.64) | 0.74 (0.62 to 0.86) |
| Central Asia | -2.52 (-2.63 to -2.41) | -2.81 (-2.91 to -2.70) | -1.99 (-2.14 to -1.85) | -2.83 (-2.93 to -2.72) |
| Central Europe | -1.05 (-1.16 to -0.95) | -1.18 (-1.30 to -1.05) | 0.23 (0.09 to 0.38) | -1.22 (-1.34 to -1.09) |
| Central Latin America | -3.02 (-3.14 to -2.89) | -3.08 (-3.22 to -2.95) | -2.23 (-2.37 to -2.09) | -3.10 (-3.24 to -2.97) |
| Central Sub-Saharan Africa | 0.65 (-0.02 to 1.32) | 0.56 (-0.09 to 1.20) | 0.89 (0.20 to 1.59) | 0.55 (-0.09 to 1.20) |
| East Asia | -1.20 (-1.54 to -0.87) | -1.29 (-1.63 to -0.95) | 1.06 (0.65 to 1.48) | -1.37 (-1.71 to -1.03) |
| Eastern Europe | -2.87 (-3.16 to -2.57) | -2.99 (-3.28 to -2.69) | -1.70 (-1.91 to -1.49) | -3.02 (-3.32 to -2.72) |
| Eastern Sub-Saharan Africa | -0.43 (-0.61 to -0.26) | -0.48 (-0.65 to -0.31) | -0.10 (-0.29 to 0.09) | -0.49 (-0.66 to -0.32) |
| High-income Asia Pacific | -4.21 (-4.44 to -3.99) | -4.36 (-4.56 to -4.15) | -2.15 (-2.38 to -1.93) | -4.59 (-4.80 to -4.37) |
| High-income North America | -1.59 (-1.64 to -1.53) | -1.78 (-1.84 to -1.73) | -0.85 (-0.93 to -0.78) | -1.86 (-1.92 to -1.80) |
| North Africa and Middle East | -1.88 (-2.03 to -1.73) | -2.00 (-2.15 to -1.86) | 0.14 (-0.01 to 0.29) | -2.07 (-2.22 to -1.92) |
| Oceania | -0.09 (-0.39 to 0.21) | -0.15 (-0.44 to 0.13) | 0.10 (-0.16 to 0.35) | -0.16 (-0.45 to 0.13) |
| South Asia | 1.47 (1.14 to 1.81) | 1.28 (0.95 to 1.62) | 1.99 (1.62 to 2.36) | 1.27 (0.94 to 1.60) |
| Southeast Asia | 2.19 (2.03 to 2.35) | 2.10 (1.96 to 2.25) | 3.22 (3.07 to 3.37) | 2.07 (1.93 to 2.21) |
| Southern Latin America | -3.35 (-3.47 to -3.23) | -3.67 (-3.79 to -3.54) | -2.59 (-2.72 to -2.46) | -3.70 (-3.83 to -3.57) |
| Southern Sub-Saharan Africa | -0.81 (-1.15 to -0.48) | -0.80 (-1.15 to -0.45) | -0.51 (-0.75 to -0.27) | -0.81 (-1.16 to -0.46) |
| Tropical Latin America | -0.37 (-0.63 to -0.11) | -0.47 (-0.73 to -0.20) | 0.36 (0.11 to 0.60) | -0.48 (-0.75 to -0.22) |
| Western Europe | -3.37 (-3.49 to -3.26) | -3.49 (-3.59 to -3.39) | -1.79 (-1.86 to -1.73) | -3.59 (-3.70 to -3.49) |
| Western Sub-Saharan Africa | 0.80 (0.71 to 0.90) | 0.72 (0.62 to 0.82) | 1.02 (0.95 to 1.09) | 0.71 (0.61 to 0.81) |

EAPC, estimated average percentage change; UI, uncertainty intervals; DALYs, disability-adjusted life years; YLDs, years lived with disability; YLLs, years of life lost;

SDI, socio‑demographic index.
